# Cracking the Skin Barrier: Models and Methods Driving Dermal Drug Delivery

**DOI:** 10.3390/pharmaceutics17121586

**Published:** 2025-12-09

**Authors:** Francelle Bouwer, Marius Brits, Joe M. Viljoen

**Affiliations:** Centre of Excellence for Pharmaceutical Sciences (Pharmacen™), Faculty of Health Sciences, North-West University, Building G16, Potchefstroom 2520, South Africa; francellebouwer@gmail.com (F.B.); marius.brits@nwu.ac.za (M.B.)

**Keywords:** dermal drug delivery, stratum corneum (SC), ex vivo skin models, reconstructed human epidermis, animal models, Strat-M^®^ membrane, diffusion cells, skin-PAMPA, tape stripping, bioequivalence assessment

## Abstract

Dermal drug delivery is a promising alternate route of drug administration, offering localized therapeutic effects, reduced systemic effects, and improved patient compliance. However, the skin’s intricate configuration, especially the stratum corneum (SC), presents formidable barriers, restricting drug permeation. This review summarizes biological, synthetic, and methodological models employed to study dermal absorption and permeability. Ex vivo human skin is a reference point, but limited availability and ethical constraints necessitate reliance on animal models, including porcine, rodent, rabbit, monkey, and even snake skin, each with unique advantages and drawbacks. Synthetic substitutes, e.g., reconstructed human epidermis and Strat-M^®^ membranes, provide reproducibility and economic practicality, though none fully mimic the barrier functions of human skin. Innovative analytical methods, including diffusion cells, skin-PAMPA, tape stripping, and advanced imaging techniques, enable quantitative, semi-quantitative, and qualitative insights into drug transport. Collectively, these tools support formulation optimization and aid regulatory bioequivalence assessments. However, challenges remain in correlating in vitro, ex vivo, and in vivo outcomes and in replicating the skin’s dynamic physiology. This review highlights current opportunities and limitations, emphasizing the need for more physiologically relevant models to advance safe, effective, and innovative dermal drug delivery systems.

## 1. Introduction

Dermal drug delivery has gained prominence in the realm of pharmaceutical sciences as an alternative to conventional administration routes, offering multiple therapeutic and practical advantages. Compared to oral administration, dermal application circumvents gastrointestinal degradation and hepatic first-pass metabolism, thereby enhancing the drug bioavailability for suitable compounds [1]. It also improves patient adherence by providing non-invasive drug delivery, prolonged release profiles, and reduced systemic side effects [2]. However, the skin serves as a highly protective barrier, which presents significant challenges to drug permeation. The stratum corneum (SC), with its highly organized lipid–protein matrix, restricts molecular transport unless compounds possess favorable physicochemical properties [3]. Beyond the SC, the epidermis and dermis also contribute to the defensive barrier characteristics, regulating transepidermal water loss and protecting against external agents [4]. This dual role, protective yet obstructive, has driven extensive research in dermal drug delivery.

**Figure 1:**
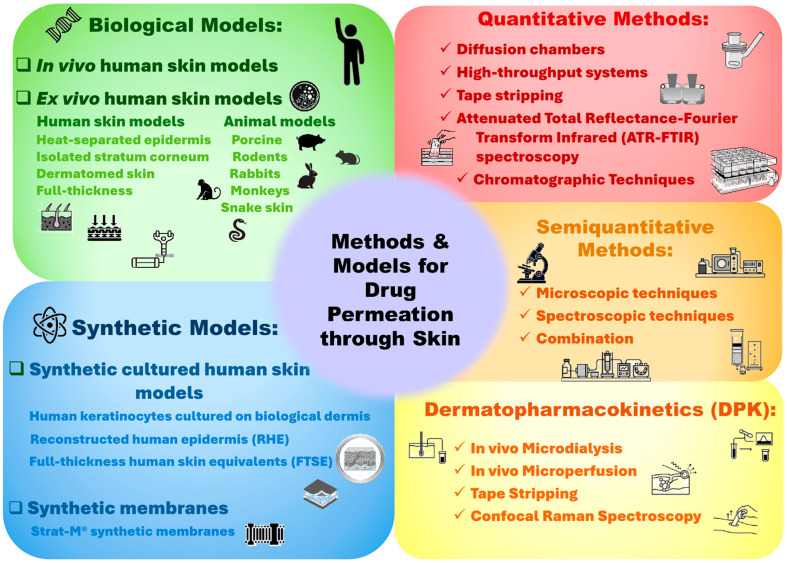
Schematic overview of the principal models and methods discussed in this review for evaluating drug permeation across the skin.

To overcome these barriers, numerous models (Figure 1) have been developed to evaluate dermal absorption and aid in formulation design. Ex vivo human skin remains the standardized model for permeation studies, offering the closest approximation to in vivo conditions. Nevertheless, limited accessibility, ethical concerns, and donor variability often restrict its use [5]. Consequently, animal models such as porcine, rodent, rabbit, monkey, and snake skin are frequently employed due to their structural similarities with human skin. However, permeability differences between these respective models complicate data correlation [6,7]. Similarly, synthetic substitutes, particularly reconstructed human epidermis (RHE) and synthetic Strat-M^®^ membranes, offer reproducibility, cost-effectiveness, and regulatory acceptance for preliminary screenings, although none fully mimic the complexity of human skin [8].

Alongside biological and synthetic models, methodological innovations have further advanced dermal drug delivery research (Figure 1). Traditional approaches, such as diffusion cells, remain the cornerstone of quantitative assessment, while high-throughput systems such as the skin parallel artificial membrane permeability assay (skin-PAMPA) are increasingly used in early-stage screening [9]. Complementary techniques, for example, tape stripping [10,11,12] and advanced microscopic and/or spectroscopic methods, provide detailed insights into drug distribution, penetration pathways, and molecular interactions within the skin layers [13,14,15,16]. Despite these advancements, bridging the gap between in vitro and/or ex vivo findings and in vivo human outcomes remains a fundamental challenge. This review highlights the strengths and limitations of current models and methodologies, underscoring the pressing need for physiologically relevant, standardized systems to accelerate the development of innovative dermal drug delivery technologies.

## 2. Dermal Delivery as an Alternative Route

Dermal drug delivery generally denotes topical and/or transdermal drug administration. Topical drug delivery refers to the application of a drug to a specific region on the body’s surface, including the nose, eyes, rectum, vagina, or skin. It offers localized therapeutic effects while minimizing or avoiding systemic exposure [17,18]. Dermal drug delivery, on the other hand, is described as “the process of mass transport of active ingredients applied on the skin to various skin strata” [19].

Over the last 30 years, delivering drugs via the skin has gained significant popularity as an alternate route of administration. This approach is increasingly preferred, particularly when the objective is to prevent complications associated with oral drug delivery [20]. Moreover, studies have shown that patients favor dosage form options that allow self-administration, are painless, minimally invasive, and reduce the need for hospital visits, thereby alleviating the healthcare strain linked to treatment [21].

One example of a skin disease that disproportionately affects under-resourced communities is cutaneous tuberculosis (CTB), classified under extrapulmonary tuberculosis (TB). CTB may often occur without pulmonary or systemic TB, although systemic TB frequently co-exists with extrapulmonary manifestations, including CTB. At present, the standard treatment of CTB mirrors that of pulmonary TB, involving combinations of oral medications administered over extended periods, often accompanied by surgical intervention. This regimen is lengthy and frequently associated with significant systemic side effects [22]. Sadly, no dermal products are currently commercially available to directly and adequately treat CTB [23,24,25]. This is only one example that stresses the need for unique dermal drug delivery systems capable of targeting the underlying infection—in this case, the bacilli within the skin lesions [26].

Developing drug delivery systems that enable site-specific targeting of one or more drugs represents a promising strategy, offering several advantages [27]. Such systems should enhance drug effectiveness while simultaneously minimizing potential side effects or toxicity, since smaller drug doses may be sufficient to achieve the same desired effect [26,28]. Delivering drugs through the dermal route provides additional benefits for treating local conditions, including improved adherence to treatment regimens, reduced risk of drug–food interactions, and avoidance of hepatic metabolism/first-pass effect [29,30].

Nonetheless, the skin functions as a highly efficient physiological barrier, which consequently restricts the penetration of many drugs in sufficient clinically relevant dosage concentrations [31,32]. In particular, the lipophilic nature of the SC, the outermost layer of the epidermis, plays a crucial role in maintaining the skin’s barrier function [32].

### The Skin: A Complex Barrier to Drug Delivery

**Figure 2:**
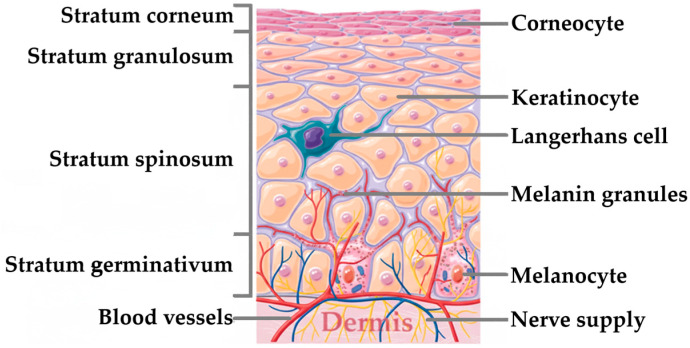
The four distinct cell layers housed within the epidermis, as generated by Servier Medical Art (SMART), licensed under CC BY 4.0.

The skin comprises three distinct layers. The hypodermis is the innermost layer of the skin, whilst the epidermis is the outermost layer. Between the hypodermis and epidermis resides the dermis. The hypodermis includes the deeper subcutaneous tissue encompassing adipose and connective tissue. Housed within the dermis are connective tissue, hair follicles, blood vessels, lymphatic vessels, and sweat glands. Conversely, the epidermis plays a role in skin tone and equips the skin with a waterproof barrier [33]. It is a stratified squamous epithelial tissue layer primarily composed of two cell types: keratinocytes and dendritic cells. Keratinocytes can be distinguished from “clear” dendritic cells as they possess intercellular bridges and a sufficient volume of cytoplasm [34]. Dendritic cells, on the other hand, are a distinguished group of specialized antigen-presenting immune cells, engaging in the initiation and regulation of the immune response. These cells retain the capacity to capture and process antigens, facilitating the transportation of the antigens from the peripheral tissue to secondary lymphoid organs, and they activate naïve T cells. Based on these properties, epidermal Langerhans cells and dermal dendritic cells are considered the key antigen-presenting cells housed in the skin [35]. Furthermore, the epidermis hosts numerous other cell populations as well, including melanocytes and Merkel cells. It is important to note that ectodermally derived keratinocytes constitute the majority of cells within the epidermis [36]. Traditionally, the epidermis is divided into four distinct cell layers based on keratinocyte morphology and position as they mature into horny cells. Thus, in order from deepest to most superficial, and as depicted in Figure 2, the epidermis comprises the basal cell layer (*stratum germinativum*), the squamous cell layer (*stratum spinosum*), the granular cell layer (*stratum granulosum*), and the cornified, or horny cell layer (*stratum corneum*) [34,37]. The two most underlying layers of the epidermis that contain living cells with nuclei are occasionally referred to as the *stratum malpighi* and/or *rete malpighi* [34].

Horny cells, also referred to as corneocytes within the cornified layer and hereinafter referred to as the SC, serve as a barrier to the lower epidermal layers. This barrier prevents excess water loss and averts intrusion by extraneous or foreign elements [38]. An extracellular lipid matrix encloses the protein-rich and minimally lipid-containing corneocytes [36]. These polyhedral-shaped horny cells that are both flat and large no longer possess their nuclei due to the process of terminal differentiation and can thus be deemed lifeless at this stage [34,36]. The physical and biological characteristics of the cells within the SC differ, depending on their location within the cell layer, as desquamation (the shedding of dead cells) is constantly occurring in the outermost skin region. For example, when comparing deeper, embedded cells to those residing in the middle region, the cells situated in the middle demonstrate a greater capability for aqueous binding due to the higher concentration of free amino acids present in the cytoplasm. Furthermore, the deeply embedded cells are densely compressed, exhibiting an increased arrangement of intercellular bonds compared to the superficial cell layers [39]. The epidermis is furthermore in a constant state of renewal, continually generating derivative structures such as pilosebaceous units (hair follicles, sebaceous glands, etc.), nails, as well as sweat glands [36].

Three distinct routes through the described skin layers exist that facilitate drug permeation of dermal drug delivery systems [40]. First, the drug substance initially penetrates the skin via the intracellular or intercellular pathways [41]. Well-hydrated keratins are crucial components of the corneocytes and constitute the primary structural elements of the epidermis, through which certain substances can pass. Moreover, mainly hydrophilic drugs more easily permeate the skin by means of the intracellular route [2]. Second, the intercellular pathway may be traversed through diffusion of a drug substance utilizing the lipid matrix of the skin, as is typically observed with lipophilic drugs [2,41]. Third, the transappendageal pathway, encompassed by hair follicles as well as sweat glands, allows for restricted drug permeation, as it is generally limited to only 0.1% of the surface of the skin [41,42]. Furthermore, pilosebaceous glands found in the transappendageal penetration route pose an additional barrier to hydrophilic substances due to the lipophilic composition of the sebum that occupies the sebaceous glands [3]. Nevertheless, this route may prove beneficial when considering the delivery of a lipophilic compound [2]. Conversely, the presence of sweat at the transappendageal site could potentially aid in the delivery of hydrophilic substances [41], although overall drug delivery is limited in the presence of sweat due to drug diffusion transpiring against the diffusion gradient [43]. Therefore, the physicochemical characteristics of a drug compound(s) are of paramount importance when designing a drug delivery system that facilitates topical and/or transdermal drug delivery [1].

However, before dermal products reach the commercial market, it is necessary to conduct a thorough evaluation of their safety and efficacy profiles to minimize the risk of severe adverse effects. Therefore, conducting, for example, skin diffusion studies can aid in establishing if the product can effectively release the included drug(s) in adequate concentrations to the skin, ensure that sufficient and safe drug concentrations permeate the skin, confirm if the product executes its intended function satisfactorily, and detect any harmful substances or metabolites that should be avoided [44].

## 3. Biological Models for Assessing Dermal Absorption

Dermal absorption should preferably be assessed under favorable conditions, with in vivo human skin considered the “gold standard” for assessing targeted drug delivery to specific areas of the skin, systemic exposure, and toxicological effects [45]. However, this approach is often impractical due to high expenses associated with human trials and ethical concerns regarding the testing of potentially harmful substances. Additionally, data obtained from in vivo test subjects can be difficult to interpret and are often subject to high variability. Therefore, accurate prediction of in vivo human outcomes requires alternative methods of assessment capable of generating reproducible and reliable data that correlate well with human skin [46].

### 3.1. Ex Vivo Human Skin Models

Extensive research employing ex vivo human skin has been conducted, largely driven by regulatory authorities aiming to establish consistent and standardized investigative methods. Noteworthy progress has been made in correlating ex vivo and in vivo dermal absorption results [5,47].

Human skin samples utilized in research are typically sourced from cosmetic procedures or cadavers, with ethical approval required in all cases. Skin samples are most frequently obtained from larger anatomical regions such as the abdomen, breast, or dorsal trunk. When designing experiments, however, it is important to realize that skin permeability varies between anatomical sites due to differences in SC thickness [48], skin hydration [49,50], and lipid content [51]. As previously mentioned, the SC constitutes the principal barrier to exogenous penetration and regulates transepidermal water loss [4,52].

Human skin can be stored at −20 °C for up to six months without compromising SC barrier function [53]. In contrast, several studies report that freezing animal skin (e.g., mouse, rat, rabbit, monkey, canine, porcine, or bovine) can alter permeation behavior, often resulting in significantly increased permeation compared to ex vivo human skin [54,55,56,57,58,59,60,61,62,63,64,65,66,67,68]. For example, Wang et al. [56] investigated the influence of different freezing–thawing procedures on the barrier function of rat and hairless mouse skin at 4 °C, ambient temperature, and 32 °C after storage at −20 °C for nine days, as well as the transdermal permeability of granisetron and lidocaine. No significant differences in steady-state flux between fresh and thawed samples were observed. However, compared with fresh skin, significant differences in the lag time for granisetron permeation were found in rat skin thawed at ambient temperature and 32 °C. Histological analysis and scanning electron microscopy revealed no obvious structural damage to the frozen–thawed skin, whereas immunohistochemical staining and enzyme-linked immunosorbent assay for the tight junction protein Cldn-1 demonstrated a significantly impaired epidermal barrier. The authors concluded that the freezing–thawing process increases the permeation rate of hydrophilic drugs in part due to functional degradation of tight junctions between SC cells [56].

Nevertheless, these effects depend heavily on the experimental design, including the type of ex vivo skin, storage temperature, storage duration, and the compound used for diffusion testing, as researchers have reported varying outcomes. Overall, studies indicate that freezing or heat exposure can significantly alter drug permeability in previously frozen animal skin due to reduced epidermal cell viability and metabolic activity [54,55,56,57,58,59,60,61,62,63,64,65,66,67,68]. Nonetheless, Barbero and Frash [58] found that cautiously handled frozen human skin remains suitable for evaluating passive diffusion, provided that cell viability and metabolism are not endpoints of interest.

Various types of membranes can be prepared from ex vivo human skin. “Full-thickness skin” includes all layers down to the dermis after removal of connective and subcutaneous adipose tissue [69]. To maintain dermal integrity and improve consistency, full-thickness skin can be sectioned using a dermatome to an approximate thickness of 500–750 μm [70]. However, the hydrated dermis can impose an additional diffusion barrier, particularly for lipophilic compounds, since it lacks the active clearance observed in living tissue. Under in vivo circumstances, absorbed molecules are rapidly removed by capillary circulation, whereas in diffusion cells this clearance is absent, mimicking a vasoconstricted state [71].

As a result, membranes comprising only the SC and viable epidermis have become widely adopted. Under these conditions, “infinite dilution” applies, as compounds that traverse the SC are instantly released into the receptor solution of the diffusion cell [71]. Isolated SC has also been employed to examine permeation [72] as well as to study SC heterogeneity and its function as a reservoir for water and various other compounds [73,74].

#### Ex Vivo Human Skin in Bioequivalence Studies

Novice topical drug formulations generally require comparative clinical endpoint studies to prove bioequivalence of the proposed model drug. While such trials provide direct in vivo data, they are often limited by high variability and low drug sensitivity resulting from intrasubject and drug-related factors. Pharmacokinetic studies may serve as an alternative, though they are only suitable for formulations exhibiting substantial systemic drug absorption [6].

As of late, alternative methodologies, including in vitro and ex vivo permeation testing, tape stripping, dermatopharmacokinetic studies, and in vivo microdialysis or microperfusion, have become increasingly adopted for bioequivalence evaluation [75]. Among these, ex vivo diffusion testing using dermatomed skin samples mounted in diffusion cells has gained considerable recognition as a reliable and predictive technique [75]. Its utility is supported by experimental evidence demonstrating strong correlations among in vitro (synthetic membrane), ex vivo (biological skin), and in vivo (human) diffusion outcomes for selected formulations [6,53]. These correlations, however, reflect experimental relationships only and do not indicate regulatory equivalence.

Establishing meaningful in vitro, ex vivo, and in vivo correlations, however, depends strongly on harmonized experimental protocols [5,76]. Although permeation testing for bioequivalence has only recently received formal recognition, its methodology has been investigated for several decades. As early as the late 1980s, the US FDA outlined critical parameters for ex vivo permeation testing, including membrane type, receptor phase composition, diffusion cell design (static or flow-through), application regimen (finite or infinite dose, and experimental temperature [77].

National drug regulatory authorities such as the United States Food and Drug Administration (US FDA), the European Medicines Agency (EMA) of the European Union, and others, along with supporting international bodies such as the Organization for Economic Cooperation and Development (OECD), have since published guidelines addressing the use of ex vivo permeation testing of topical drug products. Important to note, though, is that synthetic membranes used for permeation studies are classified as in vitro, non-biological models and serve primarily as early-stage screening tools. They do not reproduce the structural and biochemical complexity of ex vivo human skin, nor are they accepted by the US FDA as substitutes in in vitro permeation testing (IVPT), which, according to FDA terminology, refers specifically to studies conducted using excised human skin (i.e., ex vivo human skin) [78,79]. As these guidelines undergo periodic revision to incorporate evolving best practices, consulting the most current versions on the respective agencies’ websites is recommended.

A complementary technique frequently used alongside permeation testing is tape stripping, which involves the sequential removal of SC layers with adhesive tape. The US FDA has explored this method for assessing the bioavailability and/or bioequivalence of dermally applied drug formulations [80,81]. The technique is based on three key assumptions: (i) the SC represents the main rate-limiting barrier to percutaneous drug absorption; (ii) drug concentration within the SC correlates with that in the viable epidermis; and (iii) SC drug levels are more predictive of dermatological efficacy than systemic plasma concentrations [82]. Short-term exposure studies can also provide valuable insights into partitioning and diffusion dynamics, enabling prediction of overall absorption profiles [80].

Despite these advantages, tape stripping is operator-dependent, and the accuracy and reliability of the data depend on the consistency of tape application and removal as well as the analytical method used to quantify drug content [6]. Alternative methods for assessing dermal drug bioavailability and permeation are discussed in subsequent sections.

### 3.2. Animal and Cell-Based Models in Dermal Research

Biological membranes derived from various animal species are routinely employed in dermal formulation research and comparative dermatopharmacokinetics investigations. Model selection depends on the study objectives, desired data, and availability of biological material [6]. Skin samples are commonly harvested from regions such as the flank, ear, or limb. Since human skin is not always readily accessible, researchers have been prompted to use animal models for their physiological similarities to human tissue [7,58,59,60,61,62].

Despite these similarities, direct data correlation between animal and human skin remains challenging due to interspecies differences in structure and composition. Nevertheless, animal models, specifically porcine, rodent, rabbit, monkey, and snake skin, are widely used to evaluate percutaneous absorption [6,7,56,57,58,59,60,61,62,63,64,65,66]. Table 1 summarizes reported values for SC, epidermal, and whole skin thickness, as well as hair follicle densities across species, emphasizing the need for standardized reporting, as a clear inconsistency exists in the reported statistical parameters for these measurands.

*Porcine* ear skin, for example, is histologically comparable to human skin [68,90], exhibiting similar SC (21–26 μm) and epidermal thicknesses [91,92], and analogous hair follicle densities (20 versus 14–32 hairs/cm^2^, respectively) [91]. Its availability and permeability characteristics have established it as the standard surrogate in skin permeation studies [6,93]. Fourier Transform Infrared (FTIR) spectroscopy has revealed that porcine SC lipids adopt a hexagonal lattice, whereas human SC lipids predominantly exhibit an orthorhombic arrangement [94]. Regardless of these structural differences, both species share similar SC lamellar organization and lipid compositions [51,94]—mainly ceramides, cholesterol, and free fatty acids in near-equimolar ratios [95]—resulting in comparable permeability profiles for both lipophilic [96,97] and hydrophilic compounds [96,98]. Regional variations in SC lipid content, as reported by Lampe et al. [51], show a decrease from facial to abdominal to lower-limb skin, corresponding to the well-established observation that facial skin generally displays higher permeability than these other anatomical sites. Therefore, although porcine ear skin remains a widely used and valuable surrogate, care should be taken when applying its data to human dermal studies, as anatomical and lipid-related differences may still lead to variations in permeability outcomes.

*Rodent* species such as mice, rats, and guinea pigs are among the most commonly used substitutes in in vitro skin permeation studies due to their availability, relatively low cost, and manageable size. Both historical and contemporary research indicate that hairless variants of these species are often preferred for practical reasons, as they circumvent fur-related complications in that they avoid shaving-induced barrier disruption [99,100,101]. Mouse skin featured prominently in early permeation studies and continues to be used in mechanistic investigations and transgenic models [100,101]. Guinea pig skin has also been utilized and is sometimes noted for its comparatively stronger barrier function. Rat skin, in particular, is frequently selected for its closer resemblance to human skin architecture [6]. However, comparative studies consistently report that rodent skin, especially rat and mouse skin, is substantially more permeable than human skin, in some cases up to tenfold [6,102,103,104,105,106].

*Rabbit* back skin, although less commonly used than rodent and porcine skin as a substitute for ex vivo human skin, typically displays greater diffusivity compared to human skin [7]. Rabbit ear skin, with about 80 follicles/cm^2^ [107,108], has shown permeability profiles for certain drugs, such as lidocaine [107], triptorelin [109], and thiocolchicoside [110], that approximate those of human skin. Nicoli et al. [107] demonstrated its suitability for ex vivo iontophoretic studies due to electroosmotic and electrorepulsive behavior. While SC thickness is similar to human and porcine ear skin, rabbit ear skin has fewer nonpolar lipids and a thinner viable epidermis, leading to lower permeability for hydrophilic compounds (e.g., caffeine, nicotinamide) compared to porcine skin [110], while lipophilic compounds such as progesterone exhibit more comparable permeability [110]. Overall, rabbit ear skin remains more diffusive than human skin [7,107,108,109,110], but these anatomical and compositional differences should be considered when interpreting data.

*Monkey* skin has occasionally been used in dermal research due to its close phylogenetic relationship to humans and generally comparable percutaneous absorption characteristics [7]. However, its practical relevance is limited, as anatomical differences, such as denser pelage and fewer sebaceous gland openings, along with ethical and regulatory constraints, have markedly reduced its use in contemporary permeation studies [111].

*Snake* skin, more specifically, shed snake skin, has been explored in a small number of specialized investigations as a non-invasive model for studying barrier properties [6,112]. While its collection without harming the animal and its relative consistent morphology offer certain practical advantages, its application in dermal drug delivery research remains uncommon. Comparative studies have shown that permeation behavior can vary substantially between snake species, reflecting differences in lipid composition and hydration [112,113]. Although promising, the limited availability and interspecies variability of snake skin constrain its broader applicability as a standardized model [6].

## 4. Synthetic Skin Substitutes for Permeation Testing

Drug permeability is frequently assessed using synthetic substitutes for biological skin, including synthetically cultured human skin models and polymeric or lipid-based synthetic membranes. Unlike in vivo skin conditions, these in vitro membranes do not account for critical physiological processes such as metabolism, distribution, and excretion. Nevertheless, they offer distinct benefits, including structural uniformity and reproducibility, making them valuable tools for preliminary screening of numerous drug candidates [114].

### 4.1. Cultured Synthetic Human Skin

Synthetic human skin models have been under development for over three decades. Early substitutes were primarily designed to assess cutaneous irritation, involving the culture of normal human keratinocytes on biological dermis after removal of the native epidermis [115]. These early constructs evolved into reconstructed human epidermis (RHE) models, where keratinocytes are cultivated on supportive membranes [116].

Skin models are broadly categorized as RHE or full-thickness human skin equivalents (FTSE), also referred to as living skin equivalents (LSE). FTSE models are three-dimensional in vitro systems that imitate the structural and functional aspects of human skin, comprising an epidermis, dermis, and hypodermis, supported by a biomaterial scaffold that serves as an extracellular matrix [114]. While RHE models reproduce only stratified epidermis, FTSE models integrate both epidermal and dermal layers [114]. Several RHE and FTSE systems are commercially available (Table 2).

Regardless of their sophistication, no reconstructed skin model (RSM) yet fully replicates the barrier properties of human skin. Although RSMs contain cell types similar to those found in vivo, they lack essential structures such as blood vessels, hair follicles, glands, and the organized lipid network that surrounds these cells [114]. Tfayli et al. [124] analyzed the SC lipids of the RSM, EpiDerm^®^, using chromatographic and Raman spectroscopic techniques. Their findings indicated that while the overall lipid composition correlated well with human SC, differences in ceramide subclasses and lipid organization were evident. Unlike human skin, the RSM exhibited a continuous keratinized surface layer and cholesterol droplets rather than a uniform lipid distribution. These irregularities disrupt the continuity of the lipid barrier and contribute to the reduced barrier integrity observed in RSMs [124].

The lipid organization of the SC is characterized by lamellar arrangements and lateral packing. In human SC, these lamellae are categorized into long periodicity phases (LPPs) and short periodicity phases (SPPs). Studies on human skin equivalents suggest the presence of LPPs but the absence of SPPs. The barrier function of the human SC is primarily regulated by LPPs, not the SPPs. Since LPPs predominantly regulate SC barrier function, their altered distribution may increase intercellular permeability. Furthermore, the lipids in human SC equivalents typically depict a hexagonal configuration rather than the orthorhombic lipid arrangement found in native skin, potentially explaining their enhanced permeability [125].

Recent literature highlights advances in next-generation 3D in vitro skin models that incorporate both keratinocytes and fibroblasts in more physiologically structured arrangements [126,127]. Although these models expand on the capabilities of previously described reconstructed epidermal and full-thickness systems, their application in permeation studies remains constrained by differences in lipid composition and barrier organization. Ongoing improvements aim to enhance barrier relevance by adjusting ceramide subclasses, reducing unsaturated fatty acids, and increasing fatty acid chain lengths [128]. Future developments may further benefit from refining extracellular matrix formation and microenvironmental conditions, such as through the use of optimized culture media or the inclusion of additional cell types and skin appendages, to achieve a more complete representation of native skin function [114].

### 4.2. Strat-M^®^ Membranes

Many synthetic membranes employed in dermal research fail to replicate the intricate biochemical and structural complexity of the human SC, including its metabolic processes and protein–lipid interactions [129]. Strat-M^®^ membranes (EMD Millipore, MA) were specifically engineered to emulate the structural and chemical characteristics of human epidermis [129,130]. Designed to simulate both human and animal skin, these membranes have shown promise as surrogates for assessing drug permeation [130].

Although studies have indicated that Strat-M^®^ demonstrates strong correlations with human skin in terms of the overall *shape and progression* of nicotine permeation profiles under finite-dose conditions (r^2^ 0.90–0.99) [129], the absolute flux values differ by more than an order of magnitude, indicating that it reflects general permeation behavior rather than quantitative equivalence. Similar observations have been reported across multiple single-drug formulations, where a 2015 study showed that Strat-M^®^ produced permeation trends for compounds such as lidocaine, caffeine, aminopyrine, numerous parabens, and various p-aminobenzoates under infinite-dose conditions that correlated with rat and human skin data without implying numerical equivalence [131]. Collectively, these findings suggest that while Strat-M^®^ membranes can reproduce *trend-level* permeation behavior across diverse molecules, they should not be regarded as a direct or quantitatively interchangeable alternative to human skin.

**Figure 3:**
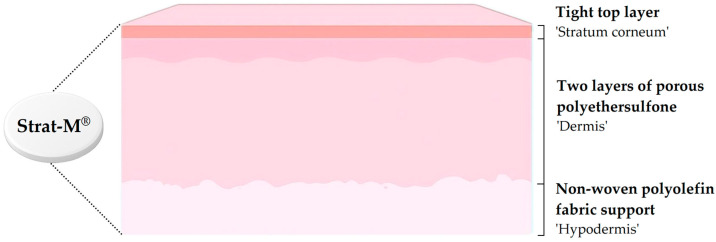
The configuration and morphology of the synthetic Strat-M^®^ membrane, adapted from Anjani et al. [132].

As summarized by Arce et al. [130], Strat-M^®^ offers advantages in product optimization, regulatory compliance, and safety assessment. Structurally, the membrane mimics the multilayered organization and lipid chemistry of human skin [130]. Each Strat-M^®^ membrane is approximately 300 µm thick, comprising a dense top layer treated with lipids and supported by two porous polyethersulfone layers resting on a non-woven polyolefin backing [129,131]. The upper layer emulates the SC, while the underlying layers replicate the epidermis and dermis [130]. The gradual increase in porosity and thickness across layers parallels the transition from epidermis to subcutaneous tissue [129], as illustrated in Figure 3. To further reproduce the hydrophobic characteristics of human skin, a proprietary lipid blend of ceramides, cholesterol, and free fatty acids is incorporated in ratios similar to those of human SC [129].

Kichou et al. [8] reported steady-state flux (J_SS_) values of resorcinol across Strat-M^®^ membranes of 41 ± 5 µg/cm^2^.h (aqueous solution), 42 ± 6 µg/cm^2^.h (hydrogel), and 40 ± 6 µg/cm^2^.h (oil-in-water emulsion). Corresponding fluxes obtained using the EpiSkin™ RHE model were significantly higher, i.e., 138 ± 5 µg/cm^2^.h, 142 ± 6 µg/cm^2^.h, and 162 ± 11 µg/cm^2^.h, while excised human skin exhibited substantially lower values of 5 ± 3 µg/cm^2^.h, 9 ± 2 µg/cm^2^.h, and 13 ± 6 µg/cm^2^.h, respectively. These findings indicate that the J_SS_ values produced by the Strat-M^®^ membranes were closer to those of excised human skin than to those obtained with EpiSkin™ RHE. Although EpiSkin™ RHE displays molecular similarity to human skin, as confirmed via spectroscopic methods, the porous, hydrophobic polymer matrix of Strat-M^®^ membranes appears to more effectively simulate the biological diffusion barrier. Similarity factor analysis (f_2_ = 80–85%) suggests that it may be reliable as an early-stage screening model; however, it cannot be considered a general alternative to human skin [8].

Owing to its structural reproducibility, ease of handling, cost efficiency, and minimal batch variability, Strat-M^®^ represents a practical and reliable alternative for evaluating dermal formulations containing single active ingredients. These advantages, combined with its correlation to human skin permeability, position Strat-M^®^ as a valuable tool for formulation design and optimization [129,130].

## 5. Methods for Evaluating Drug Permeation

Methods for evaluating drug delivery to the skin can be broadly classified into *quantitative* and *semiquantitative* or *qualitative* approaches [133]. Quantitative methods include diffusion chambers or cells, the Skin Parallel Artificial Membrane Permeability Assay (skin-PAMPA), and the tape stripping technique [133]. Skin-PAMPA is frequently employed to assess drug permeability through biological or synthetic membrane models in microplate configurations [134]. Alternatively, semiquantitative or qualitative methods encompass various microscopic and spectroscopic techniques, either individually or in combination [133].

Quantitative in vitro experiments are routinely conducted to determine the total amount of drug released on the skin and/or that permeated through biological or synthetic membranes relative to the diffusion area and acceptor chamber concentration over time [73,74,135,136,137,138,139]. In contrast, qualitative approaches primarily aim to track drug distribution across skin or artificial layers, providing spatial or relative concentration data. The combined use of both method types enhances interpretative accuracy and may facilitate regulatory approval processes [133]. Table 3 summarizes the benefits and drawbacks of each approach.

### 5.1. Quantitative Methods

Accurate dermal permeability assessment through quantitative methods depends on several experimental parameters, including solubility effects, sink conditions, incubation temperature and duration, membrane hydration, mixing speed, and dosage amount [133].

Establishing *sink conditions* for poorly soluble drugs is challenging. Acceptor media must have sufficient solubilization capacity, while maintaining the drug concentration below 10% of its solubility in the releasing matrix at the end of the experiment [140]. Proper sink conditions help maintain a stable concentration gradient and minimize backflow, i.e., the unintended movement of acceptor-phase components into the donor phase, which can reduce the driving force for permeation and lead to underestimated flux values. Adjusting the composition and pH of the acceptor and donor media, and incorporating solubilizers such as surfactants or serum albumin in the acceptor compartment, can mitigate backflow and support robust sink conditions. Serum albumin, in particular, binds lipophilic excipients to keep free drug levels low, while its large molecular weight prevents permeation through the skin [133].

*Incubation temperature* affects both the extent of drug permeation and the rheological behavior of a product or test formulation. Maintaining the structural integrity of the skin or synthetic membrane throughout the experiment is essential to avoid overestimation of absorption. Integrity is typically assessed by measuring tritiated water permeability, transepidermal water loss, or electrical resistance—each method with specific strengths and limitations. More recent approaches employ tritium-labeled internal standards measured concurrently during experimentation [141,142,143,144,145,146]. However, variability among methods and lack of harmonization persist. Standardized regulatory guidance is therefore needed to define acceptable limits and improve reproducibility of dermal formulation assessments [133].

*Skin hydration* significantly affects SC barrier function [147]. Hydrating excised skin before diffusion studies restores physiological water content, prevents collapse or stiffening of corneocytes and the SC lipid matrix, and preserves tissue microstructure [148,149]. Controlled pre-hydration markedly improves permeability consistently and comparatively between experiments using Franz diffusion cells, for example [133,150].

Adequate *mixing speed* warrants homogeneity within the acceptor compartment and minimizes boundary layer effects [140]. Controlled agitation prevents concentration gradients that could underestimate permeation rates and helps maintain sink conditions, improving reproducibility and reliability [133,150].

Experiments may employ finite or infinite dosing conditions. Infinite dosing conditions utilize formulation excess to maintain constant concentration gradients, yielding linear permeation profiles. Finite doses, in contrast, employ limited sample amounts, simulating real application conditions. The OECD guidelines recommend ≤10 µL/cm^2^ or 1–10 mg/cm^2^ for semi-solid samples under finite dosing [147], which is generally recommended [9]. Although the US FDA guidelines do not specify exact dosing ranges, it similarly emphasizes that finite doses should reflect the intended clinical application, ensuring sufficient drug permeation for measurement while maintaining realistic conditions, thus conceptually aligning with OECD recommendations [78].

Another critical consideration is *occlusion*. Occlusive conditions, achieved by covering the skin with impermeable materials, for example, films, strips, gloves, diapers, fabric garments, wound dressings, or transdermal therapeutic systems, significantly influence permeation relative to non-occluded applications [151,152,153].

#### 5.1.1. Diffusion Chambers

Diffusion experiments utilizing diffusion chambers have become the standard method for analyzing drug delivery to, and absorption through the skin, largely owing to the pioneering work of Dr. Thomas J. Franz, who invented the “Franz cell” in 1970 [154]. This method provides valuable insight into key interactions between skin, the drug substance, and the formulation or drug delivery system [154,155,156]. It closely simulates the way a formulation is applied to the skin and, consequently, how the drug is released and absorbed.

The diffusion device, commonly referred to as a diffusion chamber or diffusion cell, comprises three main components: a donor compartment/chamber for drug application, a diffusion membrane, and a receptor chamber containing the receptor medium. In principle, all diffusion cells consist of donor and receptor compartments separated by a barrier that may be a synthetic membrane, a cultured cell layer, or an ex vivo skin sample [115]. Following an experiment, the receptor medium is collected and analyzed to quantify the amount of drug that permeated the barrier [133].

Diffusion cell systems can be further classified according to their orientation (vertical or horizontal), compartment geometry and volume, and whether they are static or flow-through relative to fluid movement in the receptor compartment [115]. Among vertically oriented systems, the Franz diffusion cell (Teledyne Hanson Research, Chatsworth, CA, USA) remains the “gold standard” for transdermal drug delivery studies. It is routinely employed in both in vitro drug release and ex vivo skin permeation testing, rendering it suitable for synthetic membranes as well as excised skin samples [157,158].

**Figure 4:**
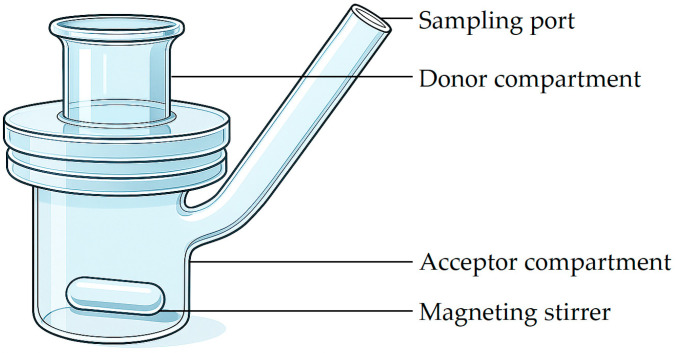
The setup of the “gold-standard” Franz diffusion cell, adapted from Nair et al. [163].

Some Franz diffusion cells are open at the top, allowing sample removal under atmospheric pressure, whereas most are sealed, which can slightly increase internal pressure and potentially yield higher diffusion values [133]. Beyond transdermal applications, Franz diffusion cells have also been adapted for nasal, corneal, and buccal drug delivery research [159,160,161,162]. A schematic representation of a conventional vertically oriented Franz diffusion cell is illustrated in Figure 4.

Traditional manually operated (“hand-sampler”) Franz diffusion chambers have largely been replaced by automated sampling systems that minimize variability and human error [133].

The µFLUX™ diffusion cell (Pion Inc., Billerica, MA, USA) is a horizontal configuration in which a synthetic membrane separates the donor and receptor compartments. This system is particularly useful for assessing the permeation and dissolution of poorly water-soluble compounds [164]. Another horizontal model, the Side-Bi-Side™ diffusion cell (PermeGear, Hellertown, PA, USA), is frequently applied in studies involving the blood–brain barrier or nasal delivery pathways [159]. The Navicyte Horizontal Diffusion Chamber System (Warner Instruments, Holliston, MA, USA) is similarly applied in toxicology and in research on transport mechanisms at air-exposed interfaces such as dermal, pulmonary, corneal, or nasal epithelia [165]. A vertically oriented Navicyte configuration has furthermore been developed, mainly for characterizing the permeability across excised tissue fragments, including intestinal, corneal, or nasal tissue samples [166,167,168].

In the static diffusion cells, the receptor solution is continuously stirred with a magnetic bar to ensure homogeneous distribution of the traversed compound. Samples are withdrawn at predetermined intervals and replaced with fresh receptor medium, either manually or through automated sampling [126].

Flow-through diffusion cells, conversely, maintain a continuous flow of receptor medium using an integrated pump. This design preserves sink conditions throughout the experiment and is particularly valuable when studying highly permeable drugs [169]. Flow-through systems also more closely simulate in vivo conditions than static cells [126]. For example, PermeGear, Inc. (Hellertown, PA, USA) supplies vertically oriented flow-through cells (“In-Line Cells”) used in a wide range of experimental studies involving synthetic membranes, excised animal or human skin, and buccal tissue samples [170,171,172].

According to the EMA [173] and US FDA [78,174] guidelines, diffusion studies are categorized as either in vitro release tests (IVRTs) or in vitro skin permeation tests (IVPTs). IVRTs employ synthetic lipid- or non-lipid-based membranes under occluded, infinite-dose conditions to assess drug release rates, typically in the microgram to milligram range. IVPTs, by contrast, use biological skin under finite, non-occluded conditions to generate drug flux profiles at lower concentrations. The EMA states that IVRTs are generally recommended for early-stage formulation development, with IVPTs subsequently utilized for formulations displaying promising release profiles [173]. The US FDA, in turn, recommends IVPT studies to compare proposed generic (test) topical products with the reference standard to demonstrate bioequivalence to the reference listed drug [78].

#### 5.1.2. Skin Parallel Artificial Membrane Permeability Assay (Skin-PAMPA)

Parallel Artificial Membrane Permeability Assays (PAMPAs) use 96-well plates with donor and receptor chambers separated by a synthetic liquid membrane. The receptor well contains buffer, while the donor well holds the test compound [114].

Originally, PAMPA models were developed to establish and predict gastrointestinal absorption [175,176,177] and later adapted for the blood–brain barrier [178]. A skin-specific version was subsequently designed [144], though it initially employed silicone oil and isopropyl myristate (IPM) as inert organic solvents. These components are not native to the skin barrier. Furthermore, the model was based on the Flynn database, which compiles data from numerous researchers under varied experimental conditions [9].

Interest in skin-PAMPAs has grown largely due to their low cost and high-throughput capability [144]. This model has since been refined through the incorporation of synthetic ceramides, cholesterol, and stearic acid to better mimic the SC [179]. Compared to diffusion cells, PAMPA offers faster, higher-throughput permeability screening, making it an increasingly attractive alternative [114]. Sinkó et al. [9] further improved the model to better predict in vitro skin penetration and to more accurately reflect SC properties [11].

As a 96-well plate-based technique, skin-PAMPA enables rapid assessment of passive permeability for diverse molecules [143]. It supports automation and has been reported to be a useful tool for preliminary absorption prediction [146] for buffer-based samples, semisolid formulations (gels, ointments, and creams) [180,181], as well as for transdermal patches [182].

Ottaviani et al. [144] evaluated 19 compounds using IPM, silicone oil, and analogous composites as liquid barriers. Permeability coefficients attained with liquid silicone membranes correlated well with data from diffusion cells using the same membrane type [183]. These silicone membranes facilitated hydrophobic compound diffusion, whereas polar molecules permeated poorly. Pure IPM did not reproduce skin permeability accurately, but a 30:70 IPM-silicone blend provided a more representative barrier, closely approximating biological skin permeability. Furthermore, the IPM component reflects the hydrogen-bonding characteristics of the SC, whereby the hydrogen bonds are primarily accepted rather than donated [144].

To improve physiological relevance, Sinko et al. [9] refined the skin-PAMPA system by developing synthetic membranes incorporating synthetic ceramides and long-chain tartaric acid diamides, analogous to biological cholesterol, free fatty acids, and ceramides in the SC. Amid the synthetic ceramides tested, C8–C18 variants produced permeability values strongly related to human skin data across 22 compounds. Nonetheless, natural skin predominantly contains longer C24–C26 ceramide chains. The skin-PAMPA model also responded to penetration enhancers such as polyethylene glycol (PEG)-400 [9]. Luo et al. [184] further showed that PAMPA differentiated between formulation types (e.g., gels and solutions) more effectively than ex vivo porcine skin or silicone membranes.

Comparative studies using commercially available patches (fentanyl, ketoprofen, rivastigmine, nicotine) revealed that both Franz cells and PAMPA produced permeation profiles exceeding manufacturer data, likely due to the so-called “putative edge effect”, in which lateral drug diffusion within the adhesive layer increases the apparent diffusion area. Reducing patch size has been suggested to minimize overestimation [182]. Rivastigmine and nicotine patches depicted similar permeation profiles in both models, though PAMPA sometimes displayed higher variability due to patch heterogeneity [182]. Likewise, Balázs et al. [180] reported strong agreement between PAMPA and vertical Franz diffusion cell results for ibuprofen formulations, with PAMPA showing lower variability, possibly because it avoids inconsistencies from ex vivo skin sample preparation [180,185].

Niacinamide permeation results correlated linearly across Franz cells and PAMPA systems using excised human or porcine skin [186]. Köllmer et al. [187] further demonstrated the affinity of the skin-PAMPA membrane for lipophilic solvents, penetration enhancers, topical emulsions, and organic receptor media additives. A modified PAMPA setup with the donor in the top plate improved sensitivity for detecting permeation.

Overall, skin-PAMPA partially replicates the SC barrier and offers a swift, cost-effective pre-screening tool for topical and transdermal formulations, enabling estimation of passive skin permeability coefficients. However, it lacks key biological intricacies such as proteins, appendages, corneocytes, native lipid composition, and epidermal architecture, limiting its application to preliminary screening rather than replacement of ex vivo skin models [114].

#### 5.1.3. Tape Stripping

**Figure 5:**
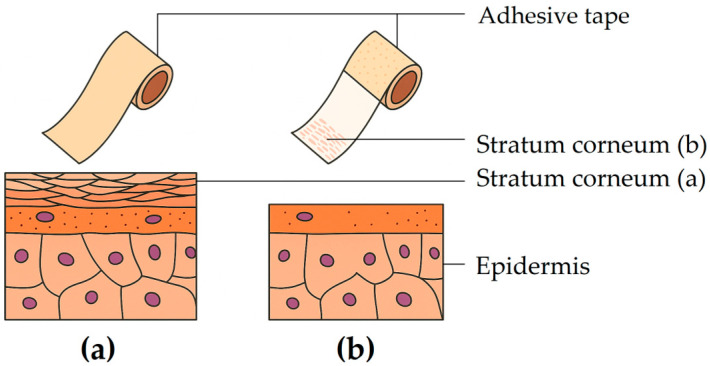
(**a**) A representation of the SC prior to tape stripping, and (**b**) the SC present on the adhesive tape, after it was removed from the epidermis by means of the adhesive tape, adapted from Ramadon et al. [189].

Adequate quantification of drug distribution across skin layers is essential for evaluating topical and transdermal drug delivery. In recent years, horizontal sectioning of the SC utilizing adhesive tape has become a standard experimental technique for determining drug concentration profiles [188]. Depending on the analytical technique, the method can yield quantitative or semiquantitative results. It is minimally invasive, as consecutive adhesive tape strips are applied to and removed from the same area on the skin sample, each removing the SC layer along with drug residues from the applied formulation. Figure 5 illustrates human SC before and after tape stripping.

Uniform tape application is crucial: strips must be pressed with consistent pressure, applied evenly, and removed at uniform speed. The removal rate also influences SC recovery—slower removal enhances adhesion and collects more material [11,190,191]. Tape stripping is performed only after sufficient incubation following topical application, and the formulation may either be wiped off or left on the surface, depending on study goals. This factor can be critical depending on the type of formulation tested and evaluated. For instance, when evaluating sunscreens, the residual surface drug content is a key indicator of formulation efficiency [11,190,191].

Beyond penetration analysis, tape stripping can quantify protein levels within skin layers and assess how drug formulations alter hydration and protein structure, including the secondary structure of keratin. It can be applied in both in vitro or in vivo on human, animal, or simulated skin models [10,11,12].

Spectroscopic methods generally yield semiquantitative data, while high-performance liquid chromatography (HPLC) provides quantitative results. Among spectroscopic tools, Attenuated Total Reflectance–Fourier Transform Infrared (ATR-FTIR) spectroscopy is most common [188]. This method measures molecular vibrations via absorption or scattering of infrared radiation, producing spectra that indicate both qualitative and quantitative information [10,11]. When combined with tape stripping, ATR-FTIR effectively detects exogenous compounds across SC layers, though interpretation can be challenging due to overlapping peaks and baseline effects. HPLC, by contrast, provides precise quantification after solvent extraction and chromatographic separation, though it is more time-consuming and destructive [133].

### 5.2. Semi-Quantitative or Qualitative Methods

Microscopic and spectroscopic methods offer key information on drug spatial distribution and penetration mechanisms within the skin. These approaches are generally non-invasive and non-destructive, unlike some in vitro techniques [13,14,15,16].

Fluorescence microscopy enables visualization of fluorescently labeled compounds within the SXC and, in some cases, deeper epidermal layers [192,193]. A more advanced form of traditional fluorescence microscopy, known as confocal microscopy, has been extensively developed over the last five decades and is able to provide higher resolution. It is now considered an indispensable research tool [10,11,194,195].

Major variants include two-photon fluorescence microscopy (2-PFM), confocal laser scanning microscopy (CLSM), and the most recently developed, confocal Raman spectroscopy. These modalities enable chemical-specific, three-dimensional imaging of biological samples under both in vitro and in vivo conditions [10,11,194,195].

#### 5.2.1. Two-Photon Fluorescence Microscopy (2-PFM)

As stated above, a valuable technique for imaging skin cells is 2-PFM, which uses a Ti-sapphire laser for excitation. When using single-photon fluorescence, a high-energy photon excites a fluorophore, raising an excited electron to a higher energy state, after which fluorescence is emitted. On the other hand, in two-photon excitation, the combined energy of two lower-energy photons is enough to excite the same electron to a higher energy level [133,196].

The setup of the two-photon microscope is similar to that of a confocal scanning microscope but differs mainly in two key ways, namely, it uses a tunable Ti-sapphire pulsed laser that emits red and near-infrared light (650–1100 nm), and it does not require pinholes in front of the detector [196]. Compared to other methods, 2-PFM has reduced total energy transmission to the test sample, which is its primary advantage. Additionally, since two-photon excitation occurs only in a very small focal volume through fluorophores, only a limited part of the sample is excited, thereby reducing the probability of photo-bleaching and tissue damage [11,196,197].

Skin specimens can be examined without the need for cryofixation or sectioning. Furthermore, infrared excitation allows for deep tissue imaging when dealing with UV-absorbing fluorophores, due to reduced scattering and absorption. However, 2-PFM has some limitations, including comparatively high costs of the laser system and the need for complex cooling equipment. It also offers slightly lower lateral resolution than some other methods, but in practice, the difference is often statistically insignificant [11,198].

#### 5.2.2. Confocal Laser Scanning Microscopy (CLSM)

As mentioned, another non-invasive technique based on fluorescence microscopy is CLSM. In recent years, CLSM has become widely accepted for imaging fluorescent model substances within the skin. It enables the investigation of skin structure without tissue sectioning and permits the analysis of how physical and chemical enhancers affect permeation [199,200,201].

CLSM has been employed in both in vitro and in vivo studies, mainly for diagnosing common skin disorders, identifying malignant lesions, and characterizing pigmentation abnormalities and keratinization [199,200,201]. It is also valuable to describe how nanoparticulate formulations enhance skin permeation. Fluorescent markers, for example, fluorescein, Nile Red, and 5-bromodeoxyuridine, can be encapsulated in nanostructured formulations, and CLSM can then be used to evaluate their penetration profiles across different skin layers as well as their associated therapeutic effects [10,11].

#### 5.2.3. Confocal Raman Spectroscopy

Spectroscopic techniques generate molecular data concerning skin structure. Among them, Raman spectroscopy, in particular, is an auspicious spectroscopic technique that identifies vibrational energy levels of molecules excited by laser light, providing data regarding the molecular structure of tissue constituents, without requiring fluorescent labels and/or chemical stains [202,203,204,205]. This makes it a powerful tool for studying structural changes in skin elements and monitoring drug permeation [206,207].

Traditionally, and as stated, drug penetration into the SC has been assessed using tape stripping, a labor-intensive and semi-destructive method that is limited to quantifying drug concentrations in the SC and is often difficult to reproduce. Then again, Raman microscopy offers a non-destructive alternative that facilitates more accurate conceptualization and deeper understanding of percutaneous drug delivery, as well as improved insight into the skin structure [202,207,208,209].

Significant technical advances in spectroscopy include custom setups and the integration of confocal microscopy with Raman spectroscopy, which enables chemically selective, non-destructive evaluation with three-dimensional spatial resolution, allowing for the simultaneous monitoring of multiple excipients. Using confocal Raman microscopy, both penetration and penetration depth of topical formulations can be assessed in vitro and in vivo [210,211].

Drug permeation can, therefore, also be studied through Raman chemical mapping. Chemical mapping is one of the techniques used in vibrational spectrometry, where a Raman spectrometer (vibration spectrometer) and microscope (optical unit) are employed to create detailed chemical maps, identifying molecular distribution within a sample at a resolution of approximately 1 μm^2^. This is because Raman spectrometry is highly selective, consisting of a fingerprint spectrum enabling apparent molecular identification. By focusing the laser on a small region of the sample, reliable chemical information can be obtained at the microscopic level [10,11,211].

The spectra compiled into a map provide extensive information about the spatial distribution of sample constituents, and multivariate data analysis can further enhance interpretation. The advantages of this approach include evaluating the distribution of physiological components of the skin and tissue, diagnosing pathological conditions, and supporting biopharmaceutical studies such as drug kinetics analysis [10,11,211].

#### 5.2.4. Attenuated Total Reflectance–Fourier Transform Infrared Spectroscopy (ATR-FTIR)

In ATR spectroscopy, the sample is positioned specifically in direct optical contact with a customized crystal known as the ATR crystal. This crystal is typically made from materials with a high refractive index, such as zinc selenide (ZnSe), which facilitates reflection at the interface [212]. The spectrometer’s infrared beam is directed onto the beveled edge of the ATR crystal by mirrors. It undergoes multiple internal reflections inside the crystal and is then redirected to the detector by an exit mirror train [212].

As stated previously, ATR spectroscopy is a rapid, surface-sensitive, and essentially non-destructive sampling approach to generate an IR spectrum. Sample preparation is minimal, provided that close optical contact between the sample and the ATR crystal is maintained [213]. A key limitation of this method, though, lies in the fragility of the ATR crystal. Crystals made of ZnSe, for example, are prone to scratching or cracking under harsh conditions, which can subsequently compromise performance and reliability [212]. To improve robustness and ease of use, more robust ATR elements have been developed, including diamond-coated and other reinforced elements. In addition, commercially available Fresnel ATR accessories with larger surface diameters (of up to 20 mm) have appeared on the market in recent years [214].

In pharmaceutics, ATR-FTIR spectroscopy is widely applied to characterize the structural and chemical characteristics of biological tissues, including the SC. Furthermore, it is also used to monitor drug release profiles from semisolid dosage forms, assess drug diffusion in, for example, polymers and films, and evaluate drug penetration into synthetic and ex vivo biological membranes. In addition, it enables the study of interactions between drugs and synthetic, semi-synthetic, or inherent molecules [213,215,216,217].

## 6. Recent Advances in Skin Models

Microfluidic skin-on-chip, organ-on-a-chip, and 3D bioprinted skin constructs represent transformative tools that replicate the complexity and dynamics of human skin for dermal drug delivery, toxicity testing, and disease modeling. Current research has increasingly focused on developing physiologically relevant systems that incorporate perfusion, immune cell interactions, and multi-layered architectures to overcome the limitations of static 2D and ex vivo models [218].

**Figure 6:**
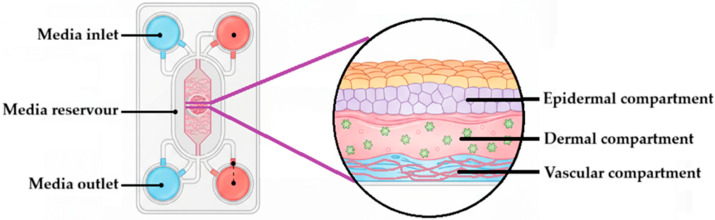
Schematic illustration of the skin-on-a-chip model. The microchip consists of three parallel channels that are separated by micro-posts and linking media reservoirs, where the central well forms the vascular compartment in the central channel.

A microfluidic skin-on-chip (Figure 6) is a laboratory apparatus designed to model human skin structure and function on a microchip. It employs microfluidic channels to regulate fluid flow and simulate the microenvironment of human skin. Typically, these devices comprise two layers, namely the epidermal and dermal layers, which are separated by a porous membrane. The design may also include blood vessels (vascular compartment) to study incidents such as inflammation, drug absorption, and immune responses without the need for animal testing [219,220]. The evolution of skin-on-chip technology has yielded systems capable of replicating full-thickness skin architecture, barrier function, and biochemical responses under dynamic flow. Rhee et al. [221] developed a perfused skin-on-chip that demonstrated near-human tissue behavior when exposed to dexamethasone and inflammatory cytokine challenges, showing excellent correlation with ex vivo biopsy responses. Similarly, Mohamadali et al. [219] introduced a pumpless skin-on-chip system powered by gravity-induced flow, maintaining skin viability and functionality for over two weeks—a significant improvement over static cultures. These studies highlight the translational potential of skin-on-chip models for evaluating drug penetration, irritation, and inflammation [222].

**Figure 7:**
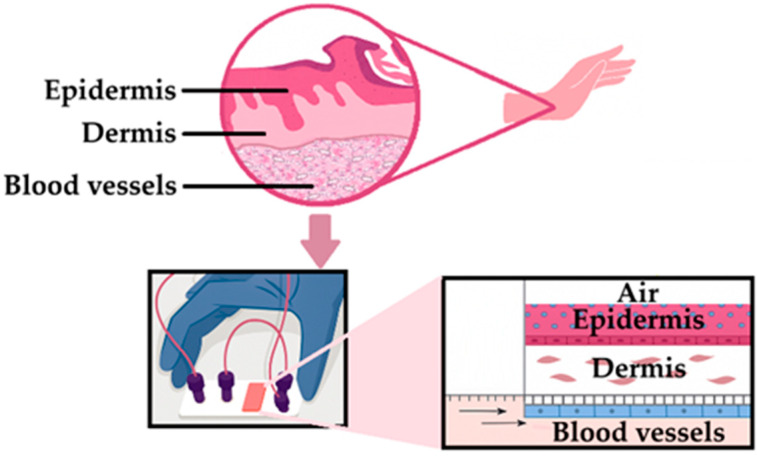
Schematic representation of the skin-organ-on-a-chip model.

Advancements in organ-on-a-chip technology have further refined these systems by integrating multiple cell types and organ interfaces to simulate immune, vascular, and metabolic interactions. A microfluidic-based skin-organ-on-a-chip platform (Figure 7) has been utilized to assess the safety of nanomaterials under dynamic flow, enabling real-time tracking of barrier disruption and immune activation [223]. This design offers enhanced predictive power for nanotoxicology and dermal exposure assessment. Furthermore, organ-on-a-chip systems have been employed for mechanistic analysis of drug permeation and for testing topically applied formulations under physiologically relevant conditions [224]. Collectively, these platforms demonstrate how fluid dynamics and co-culture techniques can recreate in vivo-like responses for pharmaceutical and cosmetic evaluation [224].

In parallel, 3D bioprinting has emerged as a powerful approach to generate architecturally precise, cell-rich constructs with controlled deposition of keratinocytes, fibroblasts, and melanocytes within extracellular matrix mimetic bioinks. Kang et al. [225] demonstrated a 3D bioprinted, vascularized, and pigmented skin model capable of simulating wound healing and pigmentation processes. Furthermore, the utility of these constructs has since expanded to disease modeling and chronic wound healing, revealing realistic cell–matrix interactions and enhanced epidermal differentiation [226]. More recently, sensor-integrated 3D bioprinted skin tissues have been developed for real-time monitoring of barrier integrity, setting a new standard for analytical precision in dermal research [227].

A particularly promising trend is the integration of 3D bioprinting with microfluidic systems, yielding hybrid, vascularized “bioprinted-on-a-chip” models. Barros et al. [228] developed a human skin-on-chip system combined with microneedling-based therapy to model drug delivery and cancer treatment, achieving spatiotemporal control of therapy delivery and perfusion [228]. These hybrid models combine the structural reliability of 3D bioprinting with the dynamic control of microfluidics, providing unparalleled opportunities for long-term, physiologically relevant skin studies. Moreover, innovations in non-absorptive polymer materials, miniaturized flow control, and automated sensor integration are expected to further enhance reproducibility and scalability [227].

Despite these advancements, challenges remain, including replicating skin appendages (i.e., hair follicles and sweat glands), achieving full innervation, and standardizing validation criteria across systems. Nevertheless, the continuous refinement of bioinks, perfusion dynamics, and microenvironmental control points to a future where skin-on-chip, organ-on-a-chip, and 3D bioprinted models will become indispensable tools in pharmaceutical and toxicological testing pipelines [218,223]. Collectively, these technologies mark a paradigm shift toward ethical, biologically relevant, and dynamically controlled approaches to dermal research.

## 7. Concluding Remarks

Dermal drug delivery represents a dynamic and rapidly evolving field in pharmaceutical research, offering distinct therapeutic and practical advantages over conventional administration routes. This review highlights the main challenges associated with the barrier function of the skin, particularly the SC, and outlines the diverse biological, synthetic, and methodological models developed to study drug permeation. Human ex vivo skin remains the benchmark model, while animal membranes and synthetic substitutes provide more accessible, reproducible, and ethically feasible alternatives. Advances such as diffusion chambers (Franz cells), skin-PAMPA, tape stripping, and high-resolution imaging and spectroscopic techniques have further broadened the toolkit for assessing drug permeation, enabling both quantitative and qualitative insights. Collectively, these models and techniques underpin the development of innovative dermal formulations and form the scientific foundation for regulatory bioequivalence testing.

Despite these advances, significant challenges persist in correlating in vitro and ex vivo findings with in vivo human outcomes, largely owing to the inherent complexity and variability of human skin. Synthetic membranes, such as Strat-M^®^ and RHE models, though promising, still fall short of fully reproducing the structural and biochemical intricacies of biological skin—particularly with respect to lipid organization and dynamic physiological processes. Future research should therefore prioritize the development of more physiologically relevant and standardized models that better mimic in vivo conditions. Moreover, establishing harmonized protocols and validation criteria across laboratories would significantly improve the reliability and comparability of permeation data.

Emerging technologies such as microfluidic skin-on-chip systems, organ-on-a-chip platforms, and 3D bioprinted skin models are transforming dermal research by replicating the physiological complexity of human skin. These advanced systems facilitate realistic evaluation of drug transport, inflammation, and disease processes under controlled, dynamic conditions. Collectively, they mark a transition toward ethical, predictive, and biologically relevant alternatives to traditional testing methods. As the demand for safe and effective dermal formulations continues to grow, the refinement of experimental models and analytical techniques will remain central to driving innovation and progress in this field.

## Figures and Tables

**Table 1:** Summary of the reported thickness of anatomical skin regions from various species frequently utilized in ex vivo dermal permeation studies [7,83,84,85,86,87,88,89].

Species	Anatomic Region	Stratum Corneum (µm)	Epidermis (µm)	Whole Skin (mm)
Human	Forearm	17	36	1.5
Human	Abdomen	6.9–9.8	64.7–95.5	1.5–3
Monkey	Abdomen	5.33 ± 0.40	17.14 ± 2.22	1.5 ± 0.7
Mouse	Dorsal	5	13	0.8
Porcine	Ear	10	50	1.3
Porcine	Dorsal	26	66	3.4
Rabbit	Ear	11.7 ± 0.5	17 ± 1.2	0.2764 ± 0.01
Rat	Dorsal	18	32	2.09
Snake	Shed (cornified layer)	10–20	N/A	N/A

N/A—not applicable, as no data was obtained.

**Table 2:** Commercially available RHE and FTSE models [112,115,116,117,118,119,120,121,122,123].

Product	Manufacturer
**RHE**	
EpiCS^®^	CellSystems, Troisdorf, Germany (HENKEL), Phenion
EpiDerm™	MatTek Corporation, Ashland, MA, USA
EpiSkin™ RHE	EpiSkin, L’Oréal Research and Innovation, Lyon, France
LabCyte EPI-MODEL 12	Japan Tissue Engineering Co. Ltd., Gamagori, Japan
LabCyte EPI-MODEL 24	Japan Tissue Engineering Co. Ltd., Gamagori, Japan
SkinEthic™ RHE	EpiSkin, L’Oréal Research and Innovation, Lyon, France
SkinEthic™ RHPE	EpiSkin, L’Oréal Research and Innovation, Lyon, France
Straticell RHE	StratiCELL, Les Isnes, Belgium
Straticell RHE-MEL	StratiCELL, Les Isnes, Belgium
**FTSE**	
EpiDerm-FT™	MatTek Corporation, Ashland, MA, USA
Apligraf^®^	Organogenesis Inc., Canton, MA, USA
Phenion^®^ FT Skin Model	Phenion, Düsseldorf, Germany
Phenion^®^ FT AGED	Phenion, Düsseldorf, Germany
Phenion^®^ FT LONG-LIFE	Phenion, Düsseldorf, Germany
StrataGraft^®^	Mallinckrodt Pharmaceuticals, Madison, WI, USA

**Table 3:** Benefits and drawbacks of frequently employed quantitative and semi-quantitative or qualitative methods for the establishment of drug penetration/permeation [131].

Method	Benefits	Drawbacks
Franz cell	Considered the “gold standard”; Recognized by drug regulatory authorities; Quantitative data is produced; Automation of the latest models; Compatible with synthetic and biological membranes; Repeated dosing is possible when employing open-top cells.	Variations exist between various laboratories; Various types of diffusion cells; Application demands practice.
Skin-PAMPA	Swift process; Numerous formulations can be studied simultaneously; Condensed area and apparatus demand; Quantitative data produced; Nearly a finite dose amount.	Mechanical process; Hand-sampling; Exclusively synthetic membranes.
Tape stripping	In vitro/ex vivo/in vivo; Only slightly invasive; Recognized by authorities; Specific layers can be evaluated.	Accurate reproducibility; Mechanical process; Hand-sampling.
Microscopy and spectroscopy	High resolution; In vitro/ex vivo/in some cases in vivo; Noninvasive; Semi-destructive; Highly selective.	Accurate reproducibility; Costly lasers required; Qualitative data produced exclusively; Extended periods required for measurement.

## Data Availability

No new data were created or analyzed in this study. Data sharing is not applicable.

## Abbreviations

The following abbreviations are used in this manuscript:

ATR-FTIR	Attenuated Total Reflectance Fourier Transform Infrared
CLSM	Confocal laser scanning microscopy
CTB	Cutaneous tuberculosis
EMA	European Medicines Agency
FTSE	Full-thickness human skin equivalent
HPLC	High-pressure performance liquid chromatography
IPM	Isopropyl myristate
IVPT	In vitro skin permeation test
IVRT	In vitro release test
J_SS_	Steady-state flux
LPP	Long periodicity phase
LSE	Living skin equivalent
OECD	Organization for Economic Co-Operation and Development
PAMPA	Parallel Artificial Membrane Permeability Assay
PEG	Polyethylene glycol
RHE	Reconstructed human epidermis
RSM	Reconstructed skin model
SC	Stratum corneum
SPP	Short periodicity phase
TB	Tuberculosis
2-PFM	Two-photon fluorescence microscopy
US-EPA	United States Environmental Protection Agency
US FDA	United States Food and Drug Administration
ZnSe	Zinc selenide
